# Editorial for the Special Issue “Applications of Optical Thin Films”

**DOI:** 10.3390/nano13081387

**Published:** 2023-04-17

**Authors:** Jin-Cherng Hsu

**Affiliations:** Department of Physics, Graduate Institute of Applied Science and Engineering, Fu Jen Catholic University, New Taipei City 242062, Taiwan

Optical thin films have been vital to enhancing optical performance for many years. The study of these films includes their design, fabrication, and metrology at the macroscopic scale, the microscopic scale, and sometimes both, depending on the requirements of critical technologies. The technologies of free-form surface optical coating, displays, optical communication, lighting, safety devices, aerospace applications, the real-time monitoring of thin films, optical coating process control, thickness distribution, etc., come under the umbrella of macro research. Microscopic studies include high-power laser coatings, biological and medical applications, ultra-lossy optical coatings, and nanostructured thin films.

Within optical research, optical films and coatings have seen significant advances since Fraunhofer discovered the antireflection effect. The wide range of applications using optical films/coatings—especially modern ones—have attracted a great deal of attention from academia; they are also an integral part of modern electronics and hold a significant share of the industry.

In this issue, several articles are related to micro studies, such as Lee et al.’s [1], wherein a metal-free and nonconjugated polymer film was developed that could play a multifunctional role in optoelectronics. High-sensitivity SERS substrates, which contain several-nanometer-thickness silver and are easily fabricated using DC magnetron sputtering, were studied by Wu et al. [2]. Spin–orbit-coupled ZnO single crystals with enhanced orientation-mediated luminescence were developed by Hassan et al. [3], and Su et al. [4] developed a Cr/n-Si Schottky junction for use in mid-infrared technology. A germanium (Ge) thin film grown using low-temperature plasma-enhanced chemical vapor deposition was developed by Lin et al. [5] to mode lock an erbium-doped fiber laser in order to generate a 1600 nm IR light. In Nicoara et al.’s study [6], crystalline PbS QDs embedded in an inorganic vitreous host matrix formed a nanocomposite material with potential for application in temperature sensor systems. On the other hand, there are also articles related to the macro level, such as Cu et al.’s [7] study, wherein a dielectric diffraction grating designed with an efficient multilayer to enhance the laser damage threshold was developed. In addition, silver-enhanced coatings with high adhesion and sulfidation resistance for telescopes with a broad spectral bandwidth from 450 nm to 1000 nm were developed by Wu et al. [8]. These articles focus on studying optical films and coatings at the macroscopic scale.

Overall, the study of optical thin films is a broad field that encompasses various applications and scales of research, and it has a significant impact on modern electronics and many other industries. This Special Issue covers applications of micro and macro optical films. We hope it can provide helpful guidance for optical applications and the development of new concepts for highly efficient multifunctional nanomaterials.
